# Obstetric determinants of preterm delivery in a regional hospital, Accra, Ghana 2016

**DOI:** 10.1186/s12884-019-2404-6

**Published:** 2019-07-15

**Authors:** Ernest Konadu Aseidu, Delia Akosua Bandoh, Donne Kofi Ameme, Priscilla Nortey, Patricia Akweongo, Samuel Oko Sackey, Edwin Afari, Kofi Mensah Nyarko, Ernest Kenu

**Affiliations:** 1 Ghana Field Epidemiology and Laboratory Training Programme, Department of Epidemiology and Disease Control, School of Public Health, University of Ghana, Legon, Accra, Ghana; 2 Namibia Field Epidemiology and Laboratory Training Programme, University of Namibia, Windhoek, Namibia

**Keywords:** Preterm delivery, Unmatched case-control, Obstetric factors

## Abstract

**Background:**

Globally, prematurity is a major determinant of morbidity and mortality contributing 30–40% of neonatal mortality. The consequences of preterm deliveries are enormous with developmental and childhood complications as well as high economic and psycho-social burden on the parents (family) and society. Some risk factors include ever having preterm delivery, multiple births and some medical conditions like sexually transmitted infection and urinary tract infections but these have not been ascertained in our study area. Much research into these risk factors is needed in Ghana. We assessed the obstetric determinants of preterm delivery.

**Methods:**

We conducted a 1:2 unmatched case-control study in Greater Accra Regional Hospital (GARH) -Ridge, a secondary referral facility in Accra, Ghana (from October, 2015 -May, 2016). A case was a mother who delivered between 28 and 36 weeks of gestation (preterm) and a control was a mother who delivered after 37 to 42 completed weeks (term). We used structured questionnaire to collect data, reviewed maternal and foetal records using a checklist. Categorical variables were analysed and expressed as frequencies and proportions. We determined the association between obstetric factors and preterm delivery with multiple logistic regression. Significance level of the strength of association was determined at 95% CI and *p*-value < 0.05.

**Results:**

We recruited 390 mothers, 130 had preterm deliveries (cases) and 260 had term deliveries (controls). Experiencing premature rupture of membrane (aOR: 2.3; 95% CI:1.0–5.5), pre-eclampsia/eclampsia (aOR: 3.4; 95% CI: 1.0–11.9) were found to be associated with preterm delivery. However, four or more ANC visit was protective factor for preterm delivery (aOR: 0.2; 95% CI: 0.1–0.4).

**Conclusion:**

Premature rupture of membrane, hypertensive complications and antepartum haemorrhage were found to be risk factors associated with preterm delivery in Ridge Hospital. Health workforce providing ANC services need to identify risk factors and refer these mothers to the doctor for early management and improved outcome decreasing preterm delivery.

## Background

According to the World Health Organization (WHO), each year, 8 million infants die before reaching their first birthday worldwide [1]. Of these, over 1 million are born preterm. Globally, 1 out of every 10 babies is born prematurely. Preterm birth is an issue of major public health concern causing deaths and high rates of morbidity and disability among survivors [2]. Preterm birth is the major cause of death during the first month of life contributing about 30–40%, and contributing 20–30% to infant and under-five mortality respectively [3]. Complications of preterm birth are the single largest direct cause of neonatal deaths. It is a significant cause of long-term loss of human potential amongst survivors. Babies who make it often face a lifetime of medical setbacks [4, 5].

Several risk factors for preterm labor and premature birth including; extremes of age (being below 19 years and being above 35 years), socio-economic status, preeclampsia, gestational diabetes, Preterm Premature Rupture of Membrane (PPROM) and foetal conditions such as foetal distress have been determined in various settings [6–9]. Other antecedents of preterm births are multiple gestations arising from the use of assisted reproductive technologies that involve implantation of multiple embryos and advanced maternal age [8–10].

To achieve the WHO-United Nations 2010 goal of reducing mortality due to preterm birth by 50% in 2025, it is essential that risk factors of preterm birth and their dynamics in different settings are well understood. Although preterm rates are higher in low-and-middle income countries including Ghana, pertinent information on risk factors of preterms are not adequately documented or readily available in the national database.

Lack of knowledge and proper documentation of the obstetric factors associated with preterm delivery in Ghana can lead to preventable preterm deliveries and its associated mortalities. The study sought to determine the obstetric factors associated with preterm delivery in Greater Accra Regional Hospital (GARH) -Ridge, Accra from October, 2015 to May, 2016.

## Methods

### Study design

A 1:2 unmatched case-control study was conducted at Greater Accra Regional Hospital, Ridge, Accra. A structured questionnaire was used to collect data on maternal and foetal records from both cases and controls to determine the obstetric factors associated with preterm delivery.

### Study setting

The Ridge Regional Hospital is a one stop shop secondary level hospital for Ghana Health Service in Greater Accra Region (GAR) and provides comprehensive Emergency Obstetric and Neonatal Care. Monthly delivery was between 700 and 800 but the proportion of preterm delivery is relatively small approximating 5.5 to 10%. Greater Accra Regional Hospital, Ridge has a Neonatal Intensive Care Unit to manage both complicated term and preterm babies and provides services under the National Health Insurance Authority (NHIA). The Greater Accra Regional Hospital (GHRH), Greater Accra Region (GAR) was selected for the study due to peculiar and relevant secondary level services it provides and also it being the main secondary referral facility within the Ghana Health Service in Greater Accra Region.

### Study population

All mothers who came to deliver in Greater Accra Regional Hospital, Ridge. This included both their regular Antenatal clinic (ANC) clients and those referred from other hospitals in Greater Accra Region to Ridge Regional Hospital for obstetric or neonatal care services. Similarly, all term and preterm babies born to these mothers- singleton and multiple outcomes-twins, triplets, quadruplets etc. were part of the study population. For multiple births, the mother –child pair was taken as the mother and the first child that was delivered by the mother.

### Case definition and selection criteria

#### Case

A mother who delivered a singleton or multiple preterm (between 28 and 37 weeks of gestation) in Greater Accra Regional Hospital, Greater Accra Region from October, 2015 to May, 2016. Eligible mothers were identified from the labour ward or theatre and then followed to Post-Natal ward.

#### Control

A mother who delivered a singleton or multiple term baby (completed 37 weeks and 42 weeks) in Greater Accra Regional Hospital, Greater Accra Region from October, 2015 to May, 2016. Two controls were selected per preterm delivery. The first control selected was a mother who delivered a term baby before the selected preterm delivery. The second control selected was a mother who delivered after the selected preterm case. Both controls were ANC attendees at Greater Accra Regional Hospital.

### Inclusion criteria

Cases – All mothers who delivered preterm babies in GARH either spontaneously or by Lower Uterine Segment Caesarean Section (LUSCS) and have been transferred to the post-natal ward.

Control – All mothers who delivered a term baby in GARH either spontaneously or by LUSCS and have been transferred to the post-natal ward.

### Exclusion criteria

Cases and Control - All mothers who delivered spontaneously or by LUSCS but were medically or psychologically unstable.

All referred mothers who had term deliveries.

### Sampling

#### Sample size

A sample of 390 mother and baby pairs comprising 130 mothers with babies who were delivered preterm (cases) and 260 mothers with babies who were delivered at term (controls) were sampled.

Calculation of the sample size was done as follows:

A 1:2 unmatched case to control ratio was used at 95% confidence interval. The sample size was calculated with the assumption that 50% of the controls and 66% of the cases were exposed to modifiable risk factors such as parity, and number of ANC visits respectively since the outcome is rare. An 80% power of effect to detect minimal odds ratio of 2 was used. Epi info Version 7 software was used in sample size calculation. Using the Fleiss formula and allowing for 10% non-response rate, the sample size was rounded of to 390 mothers comprising 130 mothers who had preterm births (cases) and 260 mothers who had term births (controls).

#### Sampling method

Within Greater Accra Regional Hospital, mothers were identified from the labour ward delivery register and then followed to the post-natal ward where the mothers are observed until they have recovered from the delivery process before discharged home.

Three categories of mothers were included here as cases. Mothers who delivered preterm babies with medical complications and so admitted to the NICU; mothers who delivered preterm babies without any medical complications but their birth weight was less than 1.5 kg and so admitted to Kangaroo Mother Care ward after observation in the NICU and mothers who delivered preterm babies without any medical complications warranting admission and their birth weight was greater than 1.5 kg and discharged home.

Controls were identified as mothers who delivered term babies.

#### Data collection technique and tools

A structured data capture sheet was designed to extract the hospital maternal health records. A face to face interview was conducted with mothers included in the study to complete the questionnaire on demographics and obstetric factors that were not documented in their records and validate what had been abstracted. The questionnaire captured antenatal visits at booking, subsequent visits- through 28 weeks, 36 weeks, and obstetric, and demographic variables of mothers and births.

#### Training of research team

The trained survey team for data collection were midwives and paediatric nursing staff recruited from their respective units in Ridge Regional Hospital where the data were collected.

#### Data processing and analysis

Data was coded and entered into Epi Info version 7 using double entry technique. Data was verified and cleaned to ensure good quality, and then exported to STATA software, Texas, USA Version 13 for analysis.

Frequencies of cases and controls were run to ascertain the completeness of each independent variable. The percentages of the cases and controls of independent variables were calculated from the frequencies. Means were calculated for continuous data.

Chi-square analysis test of proportions were done in STATA to determine statistical significance between the risk factors (Parity, Number of ANC visits, APH before, multiple birth, Mode of delivery, (P)PROM labor, Gestational Diabetes Mellitus (GDM), Pregnancy Induced Hypertension (PIH), Pre-eclampsia/eclampsia, Preterm history, Birth Interval, Previous mode of delivery) and preterm delivery. We further carried out simple logistic regressions to show their strength of associations at *p*-value of < 0.05. The variables which were significant as well as those proven to be biologically plausible in literature to be risk factors for preterm delivery were put into a multiple logistic regression model and run to determine significant risk factors.

#### Ethical consideration

Ethical approval was obtained from the Ethical Review Committee of the Ghana Health Service. Permission was obtained from the Greater Accra Regional Health Directorate, to use the Greater Accra Regional Hospital as the study site for data collection. Permission was obtained from the administration of the hospital and the respective departmental heads.

Written informed consent was obtained from mothers and confidentiality assured before the study. The participants who could not read, had the consent form read and interpreted and explained to them in the presence of an impartial witness (preferably another clinical staff who knows about the study but not a field worker). All their questions were answered to their satisfaction. Participants who agree to be part of the study were required to sign or thumbprint the consent form as an indication of their willingness to participate.

Data collected were kept confidential and used for the purpose indicated for the study. The information was securely stored with codes assigned to the participants in a file which is only accessible to the principal investigator.

## Results

The study recruited a total of 390 mothers who delivered in Greater Accra Regional Hospital comprising 130 cases (preterm deliveries) and 260 controls (term deliveries).

Out of the 130 preterm deliveries, 28% (36/130) were classified as early preterm (28 - 32 weeks gestation) and 72% (94/130) were moderate to late preterm (33–36 weeks gestation) based on the WHO classification.

### Obstetric determinants of preterm delivery

Number of antenatal Care visits (*p* < 0.001), number of babies delivered (*p* < 0.001), Pregnancy Induced hypertension (PIH) (p < 0.001), severe pre-eclampsia/eclampsia (p < 0.001), mode of delivery (*p* < 0.05), having preterm or term, Premature Rupture of Membrane (PROM) (p < 0.05), and previous mode of delivery (p < 0.05) were found to be associated with preterm delivery (Tables 1 and 2).

**Table 1:** Association between obstetric determinants and preterm delivery

Obstetric exposure	Preterm (%) *N* = 130	Term (%) *N* = 260	Total (%) *N* = 390	*P*-value
Parity				0.93
Nullip	44(33.9)	83 (31.9)	127 (32.6)	
Primip	32 (24.6)	66 (25.38)	98 (25.1)	
Multip	54 (41.5)	111 (42.7)	165(42.3)	
Number of Antenatal care visits				< 0.001
1–3	55 (43.3)	29 (11.2)	84(21.8)	
≥ 4	72 (56.7)	230 (88.8)	302 (78.2)	
Antepartum haemorrhage before				0.05
Yes	15 (12.2)	15 (6.2)	30 (8.2)	
No	108 (87.8)	229 (93.9)	337 (91.8)	
Babies delivered				< 0.001
Singleton	117 (90.0)	255 (98.08)	372 (95.38)	
Multiple	13 (10.0)	5 (1.92)	18 (4.62)	
Mode of delivery				0.02
Vaginal	44 (33.9)	119 (45.8)	163 (41.8)	
LUSCS	86 (66.2)	141 (54.2)	227 (52.3)	
(Preterm)Premature Rupture of Membrane labor				0.03
Yes	46 (35.4)	64 (24.6)	110 (28.2)	
No	84(64.6)	196 (75.4)	280 (71.8)	
Gestational Diabetes Mellitus				1.000
Yes	3 (2.3)	6 (2.3)	9 (2.3)	
No	127 (97.7)	254 (97.7)	381 (97.7)

**Table 2:** Association between Obstetric Determinants and Preterm Delivery

Obstetric exposure	Preterm (%) N = 130	Term (%) N = 260	Total (%) N = 390	*P*-value
Pregnancy induced hypertension				< 0.001
Yes	64 (49.2)	57 (21.9)	121 (31.0)	
No	66 (50.8)	203 (78.1)	269 (69.0)	
Pre-eclampsia/eclampsia				< 0.001
Yes	61 (46.9)	36 (13.9)	97 (24.9)	
No	69 (53.1)	224 (86.1)	293 (75.1)	
Preterm history				0.13
Yes	18 (13.8)	23 (8.9)	41 (10.51)	
No	112 (86.2)	237 (91.1)	349 (89.49)	
Birth Interval				0.29
< 23 months	63 (48.5)	111 (42.69)	174 (44.62)	
24–59 months	39 (30.0)	99 (38.08)	138 (35.38)	
> 60 months	28 (21.54)	50 (19.23)	78 (20.00)	
Previous mode of delivery				0.02
Vaginal	70 (78.7)	115 (65.0)	185 (69.6)	
LUSCS	19 (21.4)	62 (35.0)	81 (30.4)	

In the multiple logistic regression analysis, number of ANC visits, number of babies delivered, PROM and severe pre-eclampsia/eclampsia remained significant (Table 3 and Table 4).

**Table 3:** Obstetric Determinants associated with and Preterm Delivery

Obstetric exposure	Preterm (%) N = 130	Term (%) N = 260	cOR (95%CI)	aOR(95%CI)
Parity				
Nulliparity	44(33.9)	83 (31.9)	1	
Primiparity	32 (24.6)	66 (25.38)	0.9 (0.5–1.6)	
Multiparity	54 (41.5)	111 (42.7)	0.9 (0.6–1.5) x	
Number of Antenatal care visit				
1–3	55 (43.3)	29 (11.2)	1	1
> 4	72 (56.7)	230 (88.8)	0.2 (0.1–0.3)**	0.2 (0.1–0.4)**
Antepartum haemorrhage				
Yes	15 (12.2)	15 (6.2)	2.9 (0.8–9.7)	
No	108 (87.8)	229 (93.9)	1	
Babies delivered				
Singleton	117 (90.0)	255 (98.08)	1	1
Multiple	13 (10.0)	5 (1.92)	5.7 (2.0–16.3)**	4.9 (1.3–19.3)*
Mode of delivery				
Vaginal	44 (33.9)	119 (45.8)	1	
LUSCS	86 (66.2)	141 (54.2)	1.6 (1.1–2.6)* x	
(Preterm)Premature Rupture of membrane labor				
Yes	46 (35.4)	64 (24.6)	1.7 (1.1–2.6)*	2.3 (1.0–5.5)*
No	84(64.6)	196 (75.4)	1	
Gestational Diabetes Mellitus				
Yes	3 (2.3)	6 (2.3)	1.0 (0.2–4.1) x	
No	127 (97.7)	254 (97.7)	1	

*-*p* < 0.05 **-*p* < 0.001

**Table 4:** Obstetric Determinants associated with Preterm Delivery

Obstetric exposure	Preterm (%) N = 130	Term (%) N = 260	cOR (95%CI)	aOR(95%CI)
Pregnancy induced hypertension				
Yes	64 (49.2)	57 (21.9)	3.5 (2.2–5.4)**	
No	66 (50.8)	203 (78.1)	1	
Pre-eclampsia/ eclampsia				
Yes	61 (46.9)	36 (13.9)	5.5 (3.4–9.0)**	3.4 (1.0–11.9)**
No	69 (53.1)	224 (86.1)	1	1
Preterm history				
Yes	18 (13.8)	23 (8.9)	1.7 (0.9–3.2)	
No	112 (86.2)	237 (91.1)	1	
Birth Interval				
< 24 months	63 (48.5)	111 (42.69)	1.4 (0.9–2.3)	
24–59 months	39 (30.0)	99 (38.08)	1	
> 60 months	28 (21.54)	50 (19.23)	1.4 (0.9–2.3)	
Previous mode of delivery				
Vaginal	70 (78.7)	115 (65.0)	1	
Caesarean Section	19 (21.4)	62 (35.0)	1.99 (0.6–3.7)*	

After adjusting for all confounders, the odds of a mother with severe pre-eclampsia/eclampsia delivering a preterm was 2 times (95%CI 0.1–0.7) higher than a mother without pre-eclampsia /eclampsia

Mothers who attended antenatal clinic up to and more than four times had 0.2 (95%CI 0.1–0.4) odds of preterm delivery compared to mothers who attended ANC less than four times. The odds of a mother with multiple delivery having a preterm delivery was 4.9 times (95% CI 1.3–19.3) compared to a mother with a singleton delivery.

## Discussion

Preterm and its complications was one of the greatest challenges that prevented developing countries like Ghana from achieving the millennium development goal four (MDG 4) that focused on reducing under-five mortality [8]. This study assessed the obstetric determinants of preterm deliveries at Greater Accra Regional Hospital, Ridge, Accra. In our study, factors found to be associated with pre-term delivery and its complications were: number of ANC visits made, number of babies delivered, PROM and severe pre-eclampsia/eclampsia.

In Ghana, pre-eclampsia/eclampsia is one of the five main causes of maternal mortality with Greater Accra region recording the highest cases of pre-eclampsia in the country [11]. Three out of ten women in our study had pre-eclampsia/eclampsia. This proportion is similar to what was found by a study on hypertensive disorders in pregnancy in a referral hospital in Ghana [12] but higher than the national prevalence of almost two in ten [13]. The reason for the higher prevalence in our study could also be attributed to the fact that it is a referral facility and is located in the Greater Accra region. The odds of preterm delivery was increased by at least 3.4 times in a mother with hypertensive complication in pregnancy compared to a mother who was free of hypertensive complications in pregnancy. Our results were similar to that observed in Ethiopia where mothers who had PIH and Antepartum Haemorrhage (APH) were 2.9 times more likely to have preterm birth compared to mothers without any of the above exposures [14].

Reasons for these findings could be because; elevated blood pressure in pregnancy in itself is detrimental to the health of the mother and foetus. It compromises perfusion to the foetus and has a medical risk of cardiovascular complications for the mother. When it is complicated with urine protein and elevated uric acid level, it tips the mother into severe pre-eclampsia (imminent eclampsia) and if no action is taken eclampsia, which is life threatening for both mother and foetus ensues. This is usually unpredictable and its management is difficult, with a concomitant high mortality. Providers are therefore extremely careful in managing mothers with hypertensive complications in pregnancy. Most often, when the blood pressure become uncontrollable and/or eclampsia occurs, the quickest means of emptying the uterus become the choice for mainstay management. Thus the intervention before term accounts for the preterm delivery.

We found APH to be significantly associated with preterm delivery. The odds of preterm delivery were increased about thrice as much among mothers with APH compared to mothers who did not have APH. This is similar to a study in Nigeria where APH was observed to be significantly associated with preterm delivery [9].

Commonly the underlying causes of APH include localised vaginal bleeding and bleeding from the uterus. Localised vaginal bleeding could be as a result of trauma, spontaneous bleeding and bleeding varices. Bleeding from the uterus include placentae Previa and placentae abruption. Since bleeding from the vagina in a pregnant woman who has reached viability (> 28 weeks) requires less exploration in the vagina, a quick assessment of the mother and foetus may favour a quick emptying of the uterus. Thus, once it happens, the provider is careful not to lose either or both of the mother and baby. Mostly these obstetric indications favour a quick delivery and ultimately preterm delivery.

From our study, a mother who had Preterm Premature Rupture of Membrane (PROM) was about twice more likely to have a preterm delivery compared to a mother who did not have PROM. These findings are similar to a study in Kenya on preterm birth [10]. This is evident because anytime the membrane ruptures and the mother loses liquor, it could lead to oligohydramnios/anhydramnois with an ultimate complication of foetal distress/stillbirth or chorioamnionitis, which has far-reaching complications for the mother. This can lead to sepsis and ultimately mortality. Thus, in order to prevent these complications, providers usually intervene by either inducing them to have a vaginal delivery if the cervix is favourable or abdominally depending on whichever is the quickest way to save the mother and/or baby.

Multiple gestation, which is a grave contributor and well-established fact determining preterm delivery, was found to be positively associated with preterm delivery. This is expected because multiple gestation is typically associated with complications. In the study, there were 18 multiple gestations of which 13 were preterms and five were term.. When number of babies delivered were cross-tabulated with mode of delivery, it was noted that of the 13, only three were also delivered by caesarean section from hypertensive complication and/or PPROM. These finding are similar to earlier studies in Kenya and Ethiopia on preterm delivery [11].

Some established reasons for iatrogenic preterm delivery are maternal conditions, such as preeclampsia, and foetal distress. However, in the past decade, the number of preterm cesarean sections performed, regardless of gestational age, has increased worldwide without a similar change in maternal risk profiles [15, 16]. In this study, a mother who delivered by LUSCS was about twice more likely to be preterm compared to spontaneous delivery. These results are similar to a study on determinants of preterm delivery conducted in Ilorin, Nigeria [9].

Parity was not significantly associated with preterm delivery in this study. This is probably so because GARH is a secondary level facility and nearly all the mothers have come for high level Emergency Obstetric and Neonatal Care (EMONC) services and acts as a one stop shop facility. These findings are contrary findings of similar studies on preterm delivery, which observed that multiparity was associated to pre-term delivery [10, 17, 18]. However, a systematic review and meta-analysis on parity and preterm delivery by Shah observed that nulliparity was not significantly associated with preterm delivery [19].

In this study, a history of preterm delivery was not significantly associated with preterm delivery. The indication for the previous and the current preterm delivery is also important. Most especially if there is a predisposing obstetric indication for the previous preterm delivery and the mother did not experience the same or different obstetric indication, then, current preterm delivery is not expected. Generally, if the previous preterm delivery was spontaneous, then there is a higher odds of a current preterm delivery but in this study, an increased proportion of the preterm delivery were by caesarean section. This is supported by a study by Mazaki-Tovi and Co who observed that previous preterm delivery and recurrent preterm delivery were strongly associated with a current preterm delivery especially if it was spontaneous labour (with intact or ruptured membranes) [20]. This study findings contrasts a study done in 2014 in Kenya on preterm delivery where previous preterm birth was associated with preterm delivery [10]. The earlier the gestational age of the preterm birth, the higher the likelihood of recurrence. Therefore, counselling is advised even prior to conception to prepare the couple psychologically and efforts are being made to identify potentially treatable causes even though the attributable risk of these conditions for preterm birth is extremely low and in this study is insignificant.

### Limitations of the study

The study had some limitations; there may be differential recall bias from mothers especially from controls compared to cases. Cases are more likely to remember incidents preceding delivery compared to controls that had a normal uneventful delivery. To reduce this, records of the mothers were crosschecked from their antenatal records and important point noted done to help the mother remember what happened during her delivery.

We also observed reverse causation especially in situation where it is obvious that the exposure may be responsible for the outcome especially as related to lifestyle practises. For example, none of the mother said they smoked cigarette. Mothers are likely not to disclose certain lifestyle factors especially if it is known to be associated with preterm delivery. We made an effort to minimise this by assuring mothers of confidentiality and making them feel they were not at fault for the outcome of their pregnancy.

## Conclusion

We assessed the obstetric determinants of preterm deliveries at Greater Accra Regional Hospital, Accra. Mothers who attend ANC ≥4times are protected against present preterm delivery. However, hypertensive complications in pregnancy, antepartum haemorrhage (APH) preterm premature rupture of membrane (PPROM) were found to be determinants of preterm birth.

We recommended to ANC healthcare providers of the hospital to predict preterm delivery when a pregnant woman was found to have gestational hypertension and/or its complication, antepartum haemorrhage, preterm premature rupture of membrane and act appropriately by referring such clients to the doctor.

## Data Availability

The datasets generated and/or analysed during the current study are not publicly available due to the risk of compromising participant anonymity but are available from the corresponding author on reasonable request.

## Abbreviations

ANC: Antenatal care
aOR: Adjusted Odds Ratio
APH: Antepartum haemorrhage
AVD: Assisted Vaginal Delivery
BMI: Body Mass Index
CI: Confidence Interval
cOR: Crude Odds Ratio
EmONC: Emergency Obstetric and Neonatal Care
GARH: Greater Accra Regional Hospital
GDM: Gestational Diabetes Mellitus
LUSCS: Lower Uterine Segment Caesarean Section
NHIA: National Health Insurance Authority
NICU: Neonatal Intensive Care Unit
PIH: Pregnancy induced hypertension
PN: Post-Natal
PPROM: Preterm Premature Rapture of Membrane
PTB: Preterm Birth / Preterm delivery
SVD: Spontaneous Vertex Delivery
WHO: World Health Organization
